# Metal-dependant structural families of aminomethylphosphonic acid assemblies differentiated by ion mobility mass spectrometry and density functional theory

**DOI:** 10.1039/d5cc04906g

**Published:** 2025-10-22

**Authors:** Olivia Rusli, Haedam Mun, Marco Neumaier, Sjors Bakels, Kevin Hes, Oscar H. Lloyd Williams, Anouk M. Rijs, Junming Ho, Nicole J. Rijs

**Affiliations:** a School of Chemistry, UNSW Sydney UNSW Sydney Australia n.rijs@unsw.edu.au; b Division of Bioanalytical Chemistry, Department of Chemistry and Pharmaceutical Sciences, Amsterdam Institute of Molecular and Life Sciences, Vrije Universiteit Amsterdam 1081 HV Amsterdam The Netherlands; c Centre for Analytical Sciences Amsterdam 1098 XH Amsterdam The Netherlands; d Institute of Nanotechnology, Karlsruhe Institute of Technology Kaiserstraße 12 76131 Karlsruhe Germany

## Abstract

Herein the morphology of aminomethylphosphonic acid (AMPA)–metal aggregates are analysed by ion mobility-mass spectrometry, DFT and IRMPD spectroscopy. Matching experimental collision cross section to the DFT predicted minima allowed unambiguous assignment of [M(AMPA)(AMPA-H)]^+^ where M = Mg^2+^, Ca^2+^, and Mn^2+^. Two distinct structural families of [M(AMPA)(AMPA-H)]^+^ where M = Mg^2+^, Ca^2+^, Sr^2+^, Ba^2+^, Mn^2+^, Cu^2+^, and Zn^2+^ were differentiated by ion mobility mass spectrometry. Groupings of experimental collision cross sections were observed for a square pyramidal geometry, (M = Ca^2+^, Sr^2+^, Ba^2+^) *versus* a seesaw geometry (M = Mg^2+^, Mn^2+^, Cu^2+^, and Zn^2+^), paving the way for distinction of aggregates at the earliest stages of assembly.

## Abstract 2

Aminomethylphosphonic acid (AMPA)–metal aggregates are effectively distinguished by ion mobility-mass spectrometry, and complementary DFT, giving insight into the earliest stages of assembly.

## Graphical abstract

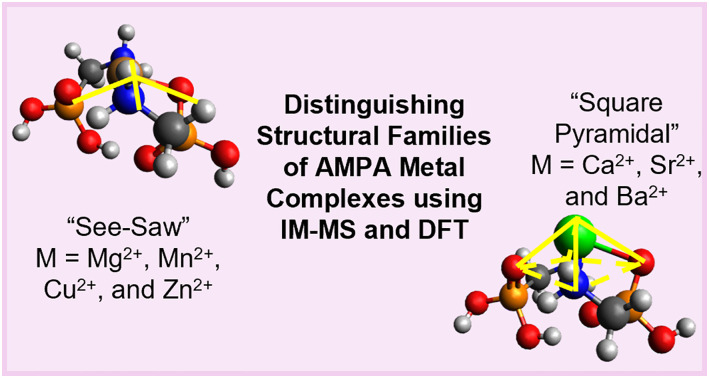

Aminomethylphosphonic acid (AMPA, Scheme 1, 1) is one of the main metabolites of glyphosate.^1^ Structurally, AMPA is an α-aminophosphonic acid and is an analogue of the simple α-amino acid, glycine (Scheme 1, 2).^2^ Due to both its environmental abundance (sourced from break down of glyphosate, and from phosphonate based detergents) and environmental persistence (much longer relative persistence than glyphosate),^3–7^ AMPA aggregation is of significant interest. The interplay between environmental and aggregation effects may play a critical role related to health and biology. For example, AMPA: has potential as a wastewater-borne marker of disease;^8^ may hamper the biotransformation potential of glyphosate;^9^ in rural waterways can change the microbial communities;^10^ exposure may influence glucose metabolism;^11^ and has been found in brain tissue of glyphosate exposed mice and may contribute to neurodegeneration.^12^

**Scheme 1:**
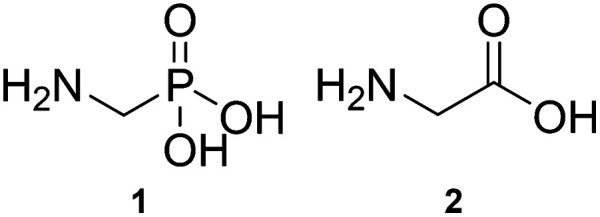
Structures of aminomethylphosphonic acid (AMPA) (1) and glycine (2).

In the environment, AMPA can sequester divalent metals, favouring Cu^2+^ > Zn^2+^ > Mg^2+^ > Ca^2+^.^13^ Synthetically, AMPA can be incorporated in metal coordination polymers, for example, the reaction between AMPA and the metal salts of Zn, Cd, Hg, Pb, Ag, Cu and Co has generated metal–phosphonate polymers (including a bimetallic metal–organic framework with Cu and Ag).^14,15^ Dimers have also been synthesised from the divalent metals copper(ii),^16^ zinc(ii),^17^ and magnesium(ii).^18^ Beyond herbicides and detergents, materials applications of AMPA are growing, for example, as an additive in photovoltaic solar cells.^19^ For synthetic and materials purposes, understanding the structural “family” of the initially formed clusters in the gas phase is a potentially convenient approach to structurally predicting the bulk compound or material.^20,21^ Structural screening approaches using ion mobility mass spectrometry could significantly speed up the discovery process, especially by avoiding the need to first crystallise synthetic targets.^20,21^

Gas phase studies also reveal unimolecular behaviours, for example AMPA anion decomposes *via* the P–C bond;^22–24^ and coordination preferences, for example divalent metals coordinate to phosphonate oxygen to form monomeric [M(AMPA-H)]^+^.^25^ Following the in depth gas phase analysis of [M(glyphosate)(glyphosate-H)]^+^ dimers, which remarkably revealed a common structural motif both between various divalent metals and between the gas phase and condensed phases,^26^ herein, the gas phase structural comparison of [M(AMPA)(AMPA-H)]^+^ dimers with divalent metal cations (M = Mg^2+^, Ca^2+^, Sr^2+^, Ba^2+^, Mn^2+^, Cu^2+^, and Zn^2+^) using cross-platform ion mobility mass spectrometry (IM-MS), combined with a robust CREST-CENSO approach to the isomeric search,^27–30^ is carried out for insight into the structural families. While not always directly comparable to the condensed phase, gas phase structures nonetheless provide fundamental insight into aggregation processes.

*ESI-IM-MS of* [*M*(*AMPA*)(*AMPA-H*)]^+^*Dimers*: Equimolar mixtures of AMPA and metal salts were analysed using electrospray ionisation (ESI) IM-MS (SI Pages S3–S5). As discussed previously,^25^ the full-scan ESI-IM-MS of AMPA and metal salts reveals formation of monomeric [M(AMPA-H)]^+^, dimeric [M(AMPA)(AMPA-H)]^+^, along with larger and higher order species (*e.g.* doubly charged). The most abundant peak in the full scan mass spectrum of all metals examined here corresponds to dimeric [M(AMPA)(AMPA-H)]^+^, with relative intensities typically more than double of the [M(AMPA-H)]^+^ ions, indicating favourable formation of the dimers.^25^

The mass-selected IM-MS arrival time distributions (ATD) of [M(AMPA)(AMPA-H)]^+^ reveal a dominant peak for all metals (Table 1, and SI, Fig. S1–S4) on both cyclic IM (cIMS) and travelling wave IM (TWIMS) platforms. Less intense secondary peaks are observed for all species except M = Ba^2+^. These additional peaks are significantly less intense and are assigned as fragments of higher order clusters for cIMS, and as contaminants for TWIMS (SI Page S9 and S10).

**Table 1:** [M(AMPA)(AMPA-H)]^+^ experimental collision cross sections (^Exp^CCS_N2_, Å^2^). Errors are ±0.3 Å^2^, (standard deviation of the measurement acquired in triplicate). The % diff. compares the ^Exp^CCS_N2_ of the cIMS and TWIMS for the dominant species

	Mg^2+^	Ca^2+^	Sr^2+^	Ba^2+^	Mn^2+^	Cu^2+^	Zn^2+^
*m/z*	245	261	309	359	276	284	285
cIMS	151.8	154.1	152.9	153.0	150.7	143.6	147.4
						(146.9)^a^	
TWIMS	152.7	156.3	156.6	156.9	151.8	150.2	149.8
% Diff.	0.4	0.9	1.6	1.7	0.5	3.0	1.1

a Shouldering peak.

Experimental CCS, denoted ^Exp^CCS_N2_, for TWIMS and cIMS were derived by calibration,^31,32^ and are in excellent agreement, a <2% difference for all metals except for M = Cu^2+^ (% diff of 3%, Table 1). [Cu(AMPA)(AMPA-H)]^+^ by cIMS has a shouldering peak (SI, Fig. S2b); this additional separation compared to TWIMS (c.f. SI Fig. S4b) may contribute to the inter-platform discrepancy. Consistently TWIMS has a larger ^Exp^CCS_N2_ than cIMS, which we attribute to differences in background molecules interacting with the analytes.^33,34^ The range of measured CCS is 143.6–156.9 Å^2^, with some structural trends apparent (Fig. 1).

**Fig. 1:**
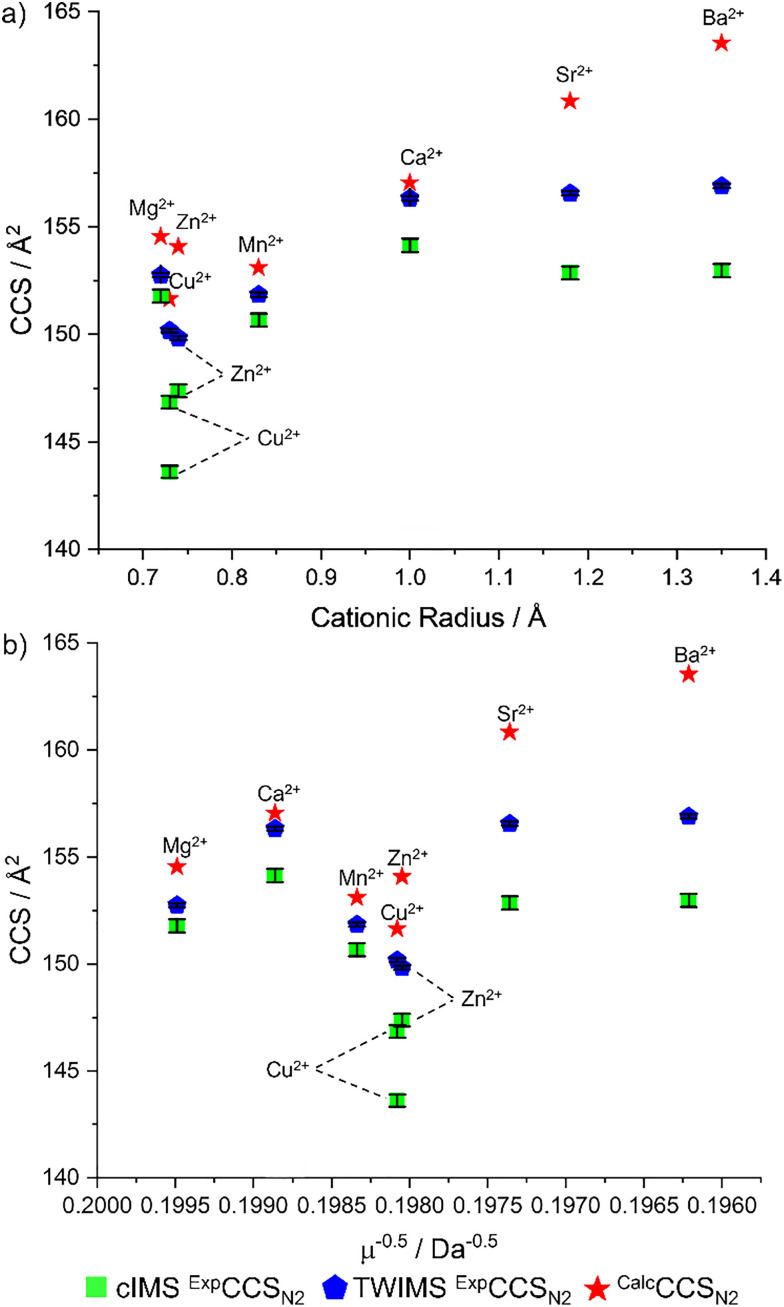
Comparison of [M(AMPA)(AMPA-H)]^+ Exp^CCS_N2_, ^Calc^CCS_N2_ and (a) cationic radius of M or (b) the reduced mass term *μ*^−0.5^ of the complex. Error bars represent the standard deviation of the CCS in triplicate (±0.1–0.3 Å^2^).

*Structural trends of measured collision cross section* (^*Exp*^*CCS*_*N2*_): It is interesting that the ^Exp^CCS_N2_ values for [M(AMPA)(AMPA-H)]^+^ when complexing with Ca^2+^, Sr^2+^ and Ba^2+^ are all essentially the same at ∼156 Å^2^, despite the significantly larger cationic radius and mass of Sr^2+^ and Ba^2+^ compared to Ca^2+^ (Table 1 and Fig. 1). The trend suggests that there is a lack of direct correlation between the metal radius and the size of the complex. It is suspected that other structural features, such as the orientation of the AMPA molecules, are the determining factor in their overall size.

Comparing the reduced mass term, *μ*^−0.5^ (where *μ* is the reduced mass for each [M(AMPA)(AMPA-H)]^+^ in N_2_ drift gas), or the cationic radius of the incorporated metal, M, to the measured CCS allows the origin of IM measured size differences to be explored. According to the low-field mobility limit (Mason-Schamp) eqn (1), the CCS should be proportional to the *μ*^−0.5^ if the CCS difference is due to mass.^35^ Likewise, size differences stemming from cationic radius of the metal will correlate with a periodic increase in radius.1
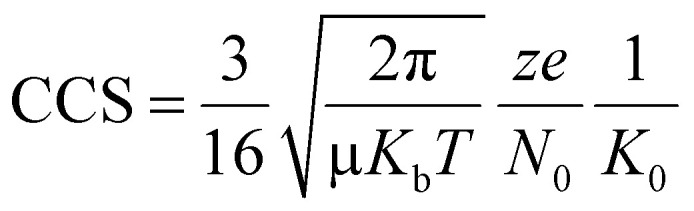


The ^Exp^CCS_N2_ for [M(AMPA)(AMPA-H)]^+^ complexes do not proportionally increase as the metal cationic radius increases (Fig. 1a). Similarly, the *μ*^−0.5^ values, do not directly correlate to the ^Exp^CCS_N2_ values (Fig. 1b). The trend for ^Exp^CCS_N2_ for [M(AMPA)(AMPA-H)]^+^ is thus neither simply proportional to the cationic radius of the metal, nor the reduced mass term *μ*^−0.5^; other factors govern the size differences.

For transition metals (M = Mn^2+^, Cu^2+^ and Zn^2+^), the ^Exp^CCS_N2_ is clustered in the range 144–151 Å^2^. The Cu^2+^ and Zn^2+^ complexes are particularly close in size. For M = Mn^2+^ the ^Exp^CCS_N2_ values are always slightly larger than for Cu^2+^ and Zn^2+^ complexes, despite the smaller mass of Mn. M = Mg also falls within this transition metal size range. For M = Mg^2+^*versus* Mn^2+^, the difference in the ^Exp^CCS_N2_ values observed is only ∼1 Å^2^ despite the difference in cationic radius of Mg^2+^ and Mn^2+^ being 0.12 Å^2^. Thus, there are two ^Exp^CCS_N2_ “clusters”; one for M = Mg^2+^, Mn^2+^, Cu^2+^ and Zn^2+^, and the other for the larger M = Ca^2+^, Sr^2+^ and Ba^2+^ suggesting the structural variation is not dependent on cation radius or mass, but rather the overall bonding and arrangement of atoms.

*Predicted* [*M*(*AMPA*)(*AMPA-H*)]^+^*structures*: For each [M(AMPA)(AMPA-H)]^+^ complex, the gas-phase structural optimisation and isomer search yielded a global minimum structure. For these structures, the metal cation coordinates to a deprotonated phosphonate oxygen, and is positioned centrally, consistent with phosphonate binding modes observed by X-ray crystallography for dimers of M = Cu^2+^, Zn^2+^ and Mg^2+^.^16–18^ In the gas phase the neutral AMPA molecule coordinates to maximise the interaction between the cation, the amine group and the phosphoryl oxygen (Fig. 2). Overall, two geometries were observed for the optimised [M(AMPA)(AMPA-H)]^+^ complexes. For complexes with metals where the metal cationic radius is less than 1 Å^2^ (M = Mg^2+^, Cu^2+^, Zn^2+^ and Mn^2+^), the [M(AMPA)(AMPA-H)]^+^ complexes adopt a see-saw geometry (Fig. 2a–d). On the other hand, when the metal cationic radius is equal to or greater than 1 Å^2^ (M = Ca^2+^, Sr^2+^ and Ba^2+^), the complex adopts a square pyramidal like geometry (Fig. 2e–g).

**Fig. 2:**
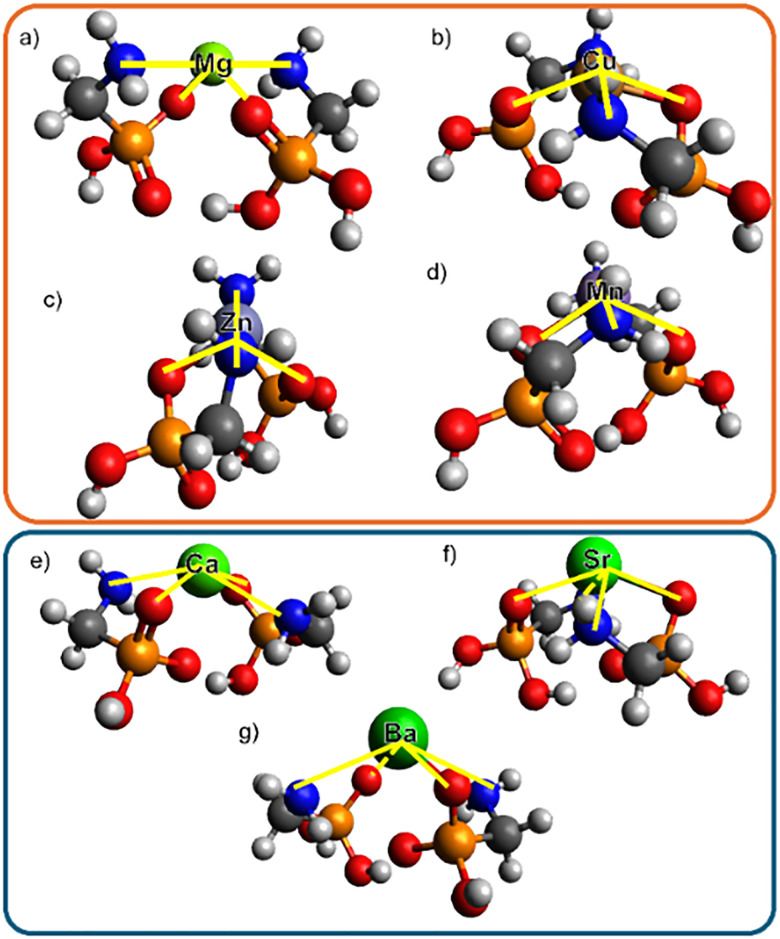
Global minimum structures (UM06/Def2TZVP) of [M(AMPA)(AMPA-H)]^+^, M = (a) Mg, (b) Cu, (c) Zn, and (d) Mn, in seesaw (orange); M = (e) Ca, (f) Sr, and (g) Ba in square pyramidal (blue).

Attempts to optimise the “alternate” metal coordination geometry for each dimer resulted to reversion to preferred geometry. Additionally, all low-lying structures also possessed the same geometry as the minima for each metal. Thus the two geometries are not competitive.

*Structural characterisation of* [*M*(*AMPA*)(*AMPA-H*)]^+^*complexes*: Experimental ^Exp^CCS_N2_ values were compared with trajectory method calculated values, denoted ^Calc^CCS_N2_, using the DFT optimised [M(AMPA)(AMPA-H)]^+^ structures (Fig. 1, red stars, see SI page S5 for CREST-CENSO and DFT method). Scaling factors were not appropriate, as the difference between ^Exp^CCS_N2_ and ^Calc^CCS_N2_ ranges between 0–7%, depending on the species (SI Pages S11–S12). While CCS is overestimated by ^Calc^CCS_N2_, trends are essentially reproduced, except for M = Sr^2+^ and Ba^2+^, which deviate from the flat trend experimentally observed for the heavier alkali earth metals (Fig. 1). The overestimate of ^Calc^CCS_N2_, may result from the non-covalent structure that determines the CCS being inadequately represented by the DFT. While ^Exp^CCS_N2_ and ^Calc^CCS_N2_ M = Ca^2+^ matches closely, for M = Sr^2+^, Ba^2+^, and also Zn^2+^, the difference between the experimental and predicted CCS values are larger than 2%, thus more experimental evidence would increase the confidence in the structural assignment of these ions. However, for M = Cu^2+^ the ^Exp^CCS_N2_ and ^Calc^CCS_N2_ difference that is larger than 2% but additional evidence from IRMPD spectroscopy lead to the confident assignment of to the predicted global minimum see saw structure (SI, Page S13). As predicted by DFT (UM06/Def2TZVP, SI, Page S5), both [Mg(AMPA)(AMPA-H)]^+^ and [Mn(AMPA)(AMPA-H)]^+^ adopt a see-saw complex geometry resulting in their very similar ^Exp^CCS_N2_ values.

Thus, the two size “clusters” experimentally observed within the CCS size trends connect to two structural families, those structures adopting the square pyramidal like geometry, with measured CCS in the range of 153–157 Å^2^ (M = Ca^2+^, Sr^2+^ and Ba^2+^), and those adopting the see saw geometry, with measured CCS in the range of 144–153 Å^2^ (M = Mg^2+^, Cu^2+^, Zn^2+^ and Mn^2+^). This helps explain that while the size of the metal determines which of the two shapes is preferred, there is no direct periodic trend resulting in a linear correlation between properties of mass or metal radii.

The gas phase structures of [Mg(AMPA)(AMPA-H)]^+^, [Ca(AMPA)(AMPA-H)]^+^ and [Mn(AMPA)(AMPA-H)]^+^ have been successfully assigned to their predicted global minimum structures from comparison of their collision cross section values without the application of a scaling factor. It was found that the metal cation coordinates to the deprotonated phosphonate group, while the neutral AMPA is positioned around the metal cation to form either a seesaw (for M = Mg^2+^ Cu^2+^, Zn^2+^ and Mn^2+^) or square pyramidal (for M = Ca^2+^ Sr^2+^ and Ba^2+^) geometry.

IM measured size trends for [M(AMPA)(AMPA-H)]^+^ complexes are mainly dictated by the overall coordination geometry and complexes that were predicted to adopt the same geometry were grouped together within a similar range of ^Exp^CCS_N2_ values. Thus, structural families of the dimers were effectively differentiated by IM-MS. This sets the scene for automated classification of assemblies at the earliest stages of aggregation using IM-MS.^21^

Conceptualization, writing – original draft, methodology, funding acquisition: OR and NJR; investigation: OR, HM, MN, SB, KH, AMR, and NJR; formal analysis: OR, HM, OHLW, MN, SB, KH, and NJR; visualization: OR, HM, OHLW, SB, NJR; OR and NJR; writing – review & editing: OR, HM, OHLW, MN, SB, KH, AMR, JH, and NJR resources, supervision: AMR, JH, and NJR.

We acknowledge the following funding, NJR: Australian Research Council (DE170100677); AMR: Dutch Research Council *via* VICI (VI.C.192.024), Aspasia (015.015.009), and KIC Key Technologies (KICH1.ST01.20.041); OR: UNSW University International Postgraduate Award, Chemistry Postgraduate Travel Award, and Development, Research and Development Grant. Access: Australian Government National Computational Infrastructure *via* UNSW Resource Allocation Scheme (gy60) and NCMAS (cw7); UTS Proteomics Core Facility. Thanks to Maria Pettyjohn, River Pachulicz, Tara Pukala, Boris Ucur, Shane Ellis, Matt Padula and Frank Hennrich.

## Conflicts of interest

There are no conflicts to declare.

## Supplementary Material

CC-061-D5CC04906G-s001

## Data Availability

The data supporting this article have been included as part of the supplementary information (SI). Supplementary information: methods, spectra, analysis and citations. The DFT optimised coordinates are available at: https://doi.org/10.5281/zenodo.16417310. See DOI: https://doi.org/10.1039/d5cc04906g.
