# Supplementary material for: Metal-dependant structural families of aminomethylphosphonic acid assemblies differentiated by ion mobility mass spectrometry and density functional theory
Source: Chem Commun (Camb). 2025 Oct 22;61(94):18621–4. doi: 10.1039/d5cc04906g (PMC12573075; doi:10.1039/d5cc04906g)
Supplement: CC-061-D5CC04906G-s001 [file CC-061-D5CC04906G-s001.pdf]

Supplementary Information (SI) for ChemComm.
This journal is © The Royal Society of Chemistry 2025

Metal-Dependant Structural Families of Aminomethylphosphonic Acid Assemblies Differentiated by Ion Mobility Mass Spectrometry and Density Functional Theory

*Olivia Rusli,[a] Haedam Mun,[a] Marco Neumaier,[d] Sjors Bakels,[b,c] Kevin Hes,[b,c] Oscar H. Lloyd Williams,[a,#] Anouk M. Rijs,[b,] Junming Ho,[a c] and Nicole J Rijs[a]**

[a.] *School of Chemistry, UNSW Sydney, Sydney, NSW, 2052, Australia*
[b.] *Division of Bioanalytical Chemistry, Department of Chemistry and Pharmaceutical Sciences, Amsterdam Institute of Molecular and Life Sciences, Vrije Universiteit Amsterdam, 1081 HV Amsterdam, The Netherlands*
[c.] *Centre for Analytical Sciences Amsterdam, 1098 XH Amsterdam, The Netherlands*
[d.] *Institute of Nanotechnology, Karlsruhe Institute of Technology, Kaiserstraße 12, 76131 Karlsruhe, Germany*

Now at: Department of Infectious Diseases, The University of Melbourne, Parkville, Australia

Corresponding Author(s)* E-mail: n.rijs@unsw.edu.au

Supporting Information

DFT optimised coordinates are available at: https://doi.org/10.5281/zenodo.16417310

Table of Contents

Methods S3

Arrival Time Distribution of Mass-selected [M(AMPA)(AMPA-H)]$^+$ on cIMS S7

Arrival Time Distribution of Mass-selected [M(AMPA)(AMPA-H)]$^+$ on TWIMS S8

Discussion: Extra Peaks from High Order (multiply charged) cluster fragmentation S9

Discussion: Scaling Factors and the Comparison with Glyphosate Dimers S11

Discussion: IRMPD Spectroscopy S13

References S15

Methods

Chemicals Aminomethylphosphonic acid (99%), poly-DL-alanine (MW 1,000 - 5,000), metal nitrate salts including: magnesium nitrate hexahydrate (99.99%); copper(II) nitrate hemi(pentahydrate) (≥99.99%); strontium nitrate (99.99%); barium nitrate (99.99%), calcium chloride dihydrate (≥99 %), zinc sulfate monohydrate (>99.9%), manganese(II) phtalocyanine, and barium dichloride dihydrate (≥99%) and zinc nitrate hexahydrate (≥ 99.0%) were purchased from Sigma Aldrich. Calcium nitrate hydrate (99.99%) and manganese (II) nitrate hydrate (99.99%) were sourced from Alfa Aesar. Magnesium acetate-4-hydrate (≥99.5%) was purchased from Riedel-de-Haën. Methanol (HPLC Grade) was obtained from Honeywell. Milli-Q water was obtained from a Milli-Q purification system with a resistivity of 18.2 MΩ·cm at 25 °C. All reagents and solvents were used as supplied without further purification.

Vial and Sample Preparation To remove sodium ions commonly found in glass vials, the vials were soaked in 10% acetic acid overnight. They were then rinsed with water, followed with methanol and air dried prior to use.

A 1 mM aminomethylphosphonic acid solution and 10 mM stock solutions of the respective metal salts in 1:1 MeOH/H_2O were combined in a 9:1 ratio, resulting in sample solutions of ~1mM. The solutions were colourless with all components fully dissolved. Sample solutions were prepared immediately prior to analysis in desalted vials. The pH of the solutions ranged from 4–5, with no pH adjustment carried out.

Ion Mobility Mass Spectrometry (IM-MS) Three different IM-MS platforms with different capabilities, namely a Synapt XS (Waters, Milford, MA, USA), a SELECT SERIES Cyclic IMS (Waters, Milford, MA, USA) and the Photo-Synapt (a modified Synapt G2, as previously described[22]), were used to investigate the $[M(AMPA)(AMPA-H)]^+$ dimers.

The Synapt XS (denoted TWIMS) and the Cyclic IMS (denoted cIMS) were used to derive rotationally averaged collision cross section values in nitrogen, $^{Exp}CCS_{N2}$. Cross-platform measurement of CCS increases the robustness of CCS values. These instruments have different configurations and resolving power. As clusters in the full scan mass spectra are made up of repeated molecular subunits,[1] mass-selection of the ions of interest pre-mobility was crucial to reduce the interference of post-mobility dissociation products.[2]

TWIMS Conditions The sample was infused directly via a syringe pump connected to an ESI source at a flow rate of 10 µL/min. All spectra were acquired at an *m/z* range of 50-2000 for 1 minute. The instrument was mass and mobility calibrated on the day of use using Waters Major Mix IMS/ToF calibration kit. As mentioned, pre-mobility mass-selection eliminates interferences on the mobilogram coming from peaks resulted from post-mobility dissociation. The instrument was operated in positive ion mode using N_2 as the drift gas, with the following conditions: capillary voltage: 3kV; source temperature: 100 °C; cone voltage: 40 V; source offset: 30 V; cone gas flow rate: 50 L/h; desolvation temperature: 350 °C; desolvation gas flow rate: 600 L/h; IMS wave height: 40 V and IMS wave velocity: 183 m/s. Data was analysed using Waters DriftScope and MassLynx. CCS values for the mass-selected complexes of interest were calculated based on a polyalanine calibration, as previously outlined.[3]

cIMS Conditions The mass-selected experiment above was repeated with cIMS for improved separation. The instrument was operated in positive ion mode and the sample was infused directly using a syringe pump connected to an ESI source at a flow rate of 10 µL/min. N_2 was used as the drift gas and the mass spectra were acquired at an *m/z* range of 50-2000 for 1 minute. The instrument was operated using the following conditions: capillary voltage: 2kV; source temperature: 100 °C, cone voltage: 40V; source offset: 10V; cone gas flow rate: 0 L/h; desolvation temperature: 250 °C; desolvation gas flow rate: 800 L/h; IMS wave height: 22 V and IMS wave velocity: 375 m/s. Data was acquired after 1 and 2 cycles around the mobility cell, a total time of 22 and 44 ms, respectively. As 2 cycles did not yield further separation, 1 cycle was used. The CCS was calibrated using Agilent tuning mix according to the equation reported by Hennrich *et al*.[4]

IM-IRMPD Spectroscopy Conditions The Photo-Synapt modifications enable ion mobility slicing and, after ion mobility, trapping of the ions in hexapole pin traps.[5] This, together with optical access for laser systems, allows for infrared spectroscopic investigations of mass- and mobility selected ions. The experiments were conducted using an MSquared FireFly infrared laser. In short, the intensity of precursor and fragment ions was recorded as a function of the wavelength, after which they were converted to IRMPD yield via $-\ln\left(\frac{I_{precursor}}{\sum I_{fragments} + I_{precursor}}\right)$. The samples were directly infused into the ESI source using a syringe pump at a flow rate of 3 µL/min with N_2 as the drift gas. The instrument was operated using the following conditions: capillary voltage: 2 – 2.4 kV; source temperature: 80 °C, cone

voltage: 40-45V; source offset: 10V; cone gas flow rate: 0 L/h; desolvation temperature: 150 °C; desolvation gas flow rate: 500 L/h; IMS gas flow rate: 50 ml/min; IMS wave height: 20 V and IMS wave velocity: 490 m/s. Data was acquired for *m/z* range of 50-1000, and IR absorbances were investigated in the 2700-3700 cm^{-1} range with the specifics for each complex detailed in the Supporting Information, page S3.

Data analysis was conducted using MassLynx and Origin, where averaging and plotting was conducted. The final experimental IR spectra are averages of 5 separate spectra.

Computational Methods and Data Analysis DFT calculations were performed using the Gaussian 16 and ORCA programs.[6–8] The CREST-CENSO calculations were conducted with five different starting geometries (representing different binding modes between the AMPA molecules and the metal cation) per complex to generate an ensemble of low energy conformers using GFN2-xTB.[9–12] Some of these CENSO run were incomplete due to SCG convergence failure, especially where M = Mn^{2+} and Cu^{2+} as these have unpaired electrons. The resulting conformers from the ensemble were reoptimized with B3LYP-D3(BJ)/6-31+G(d) for M = Mg^{2+}, Ca^{2+}, Mn^{2+}, Cu^{2+} and Zn^{2+} and B3LYP-D3(BJ)/defTZVPP for M = Sr^{2+} and Ba^{2+}. Single point calculations at revDSD-PBEP86-D4/def2TZVPP then identified the lowest energy (global minimum) structure, as this level of theory delivers conformational energies with accuracy comparable with CCSD(T)/CBS.[13]

For consistency and comparisons with previous predicted ion mobility collision cross sections[14] and to allow for accurate vibrational analysis for predicted IR structures using the available benchmarked vibrational frequency scaling factor,[15] the global minimum structures, along with key alternative structures for comparison as mentioned in the text, were reoptimized and frequency analysis was carried out at the (U)M06/Def2TZVP level of theory.[9–11,16] The gas phase structures optimized at the (U)M06/def2TZVP level of theory, along with calculated NPA partial charge,[17] were used as inputs for CCS calculation using the trajectory method in the IMoS v1.12 package using the default parameters in N_2.[18] The trajectory method (TM) approximates CCS by simulating the trajectories of buffer gas molecules into the analyte molecule allowing the prediction of its size and shape. This method considers both the short- and long-range interactions between the analyte molecule and the buffer gas molecules, resulting into a more accurate approximation of the molecule's true size and shape,[19] making TM the

gold standard method in CCS modelling. The outputs from the trajectory method carried out in N_2 gas are denoted $^{Calc}CCS_{N2}$.

All structures, predicted frequencies for IR spectra and energetics mentioned in the text are at the (U)M06/def2TZVP level of theory. Predicted IR spectra frequencies were scaled by a factor of 0.95 as benchmarked by Truhlar *et al.*[15]

Table S1. The relative energy difference between optimised (B3LYP-D3(BJ)/6-31+G(d) for M = Mg^{2+}, Ca^{2+}, Mn^{2+}, Cu^{2+} and Zn^{2+} and B3LYP-D3(BJ)/defTZVPP for M = Sr^{2+} and Ba^{2+}) global minimum structure and other conformers for each metal complex studied.

M	File Name	ZPE / Hartree	Correction Gibbs / Hartree	Gibbs / Hartree	Relative Gibbs / kJ mol^{-1}
Mg^{2+}	AMPA.a.Mg.CONF1.B3LYP	-1525.0923	0.1335	-1524.9588	0.0000
	AMPA.b.Mg.CONF1.B3LYP	-1525.0517	0.1265	-1524.9253	87.9676
	AMPA.b.Mg.CONF3.B3LYP	-1525.0505	0.1264	-1524.9240	91.1721
	AMPA.b.Mg.CONF2.B3LYP	-1525.0520	0.1293	-1524.9227	94.7231
	AMPA.b.Mg.CONF4.B3LYP	-1525.0478	0.1286	-1524.9192	103.7532
Ca^{2+}	AMPA.a.Ca.CONF1.B3LYP	-2002.4794	0.1300	-2002.3494	0.0000
	AMPA.a.Ca.CONF2.B3LYP	-2002.4763	0.1300	-2002.3463	8.1970
	AMPA.b.Ca.CONF1.B3LYP	-2002.4664	0.1323	-2002.3341	40.1851
	AMPA.b.Ca.CONF3.B3LYP	-2002.4644	0.1316	-2002.3328	43.6710
	AMPA.b.Ca.CONF6.B3LYP	-2002.4636	0.1317	-2002.3319	45.9647
	AMPA.b.Ca.CONF2.B3LYP	-2002.4636	0.1317	-2002.3319	46.0335
	AMPA.b.Ca.CONF4.B3LYP	-2002.4624	0.1316	-2002.3308	48.8577
	AMPA.b.Ca.CONF5.B3LYP	-2002.4619	0.1315	-2002.3304	50.0667
Sr^{2+}	AMPA.a.Sr.CONF3.B3LYP	-1355.9173	0.1278	-1355.7895	0.0000
	AMPA.a.Sr.CONF1.B3LYP	-1355.9173	0.1279	-1355.7894	0.2147
	AMPA.a.Sr.CONF2.B3LYP	-1355.9173	0.1279	-1355.7894	0.2452
	AMPA.a.Sr.CONF6.B3LYP	-1355.9146	0.1277	-1355.7869	6.6220
	AMPA.a.Sr.CONF4.B3LYP	-1355.9143	0.1282	-1355.7861	8.8723
	AMPA.a.Sr.CONF5.B3LYP	-1355.9143	0.1282	-1355.7861	8.8872
	AMPA.b.Sr.CONF1.B3LYP	-1355.8933	0.1255	-1355.7678	56.9395
	AMPA.b.Sr.CONF2.B3LYP	-1355.8928	0.1254	-1355.7674	57.9604
	AMPA.b.Sr.CONF4.B3LYP	-1355.8916	0.1249	-1355.7667	59.7722
	AMPA.b.Sr.CONF3.B3LYP	-1355.8915	0.1249	-1355.7666	59.9223
Ba^{2+}	AMPA.a.Ba.CONF1.B3LYP	-1350.6710	0.1264	-1350.5447	0.0000
	AMPA.a.Ba.CONF2.B3LYP	-1350.6710	0.1264	-1350.5447	0.0155
	AMPA.a.Ba.CONF4.B3LYP	-1350.6687	0.1260	-1350.5426	5.3556
	AMPA.a.Ba.CONF3.B3LYP	-1350.6688	0.1263	-1350.5425	5.7425
	AMPA.b.Ba.CONF1.B3LYP	-1350.6614	0.1250	-1350.5365	21.5516
Mn^{2+}	AMPA.a.Mn.CONF1.B3LYP	-2475.7798	0.1298	-2475.6501	0.0000
	AMPA.b.Mn.CONF1.B3LYP	-2475.7746	0.1277	-2475.6470	8.1338
Cu^{2+}	AMPA.a.Cu.CONF1.B3LYP	-2965.1882	0.1315	-2965.0567	0.0000
	AMPA.c.Cu.CONF1.B3LYP	-2965.1735	0.1286	-2965.0449	31.0108
Zn^{2+}	AMPA.a.Zn.CONF1.B3LYP	-3104.1003	0.1324	-3103.9680	0.0000
	AMPA.a.Zn.CONF2.B3LYP	-3104.1008	0.1331	-3103.9677	0.6791
	AMPA.a.Zn.CONF3.B3LYP	-3104.0973	0.1323	-3103.9649	7.9455
	AMPA.b.Zn.CONF1.B3LYP	-3104.0594	0.1264	-3103.9330	91.8803
	AMPA.b.Zn.CONF2.B3LYP	-3104.0591	0.1261	-3103.9330	91.9515
	AMPA.b.Zn.CONF4.B3LYP	-3104.0542	0.1244	-3103.9298	100.2745
	AMPA.b.Zn.CONF3.B3LYP	-3104.0547	0.1263	-3103.9284	103.7977

Arrival Time Distribution of Mass-selected [M(AMPA)(AMPA-H)]$^+$ on cIMS

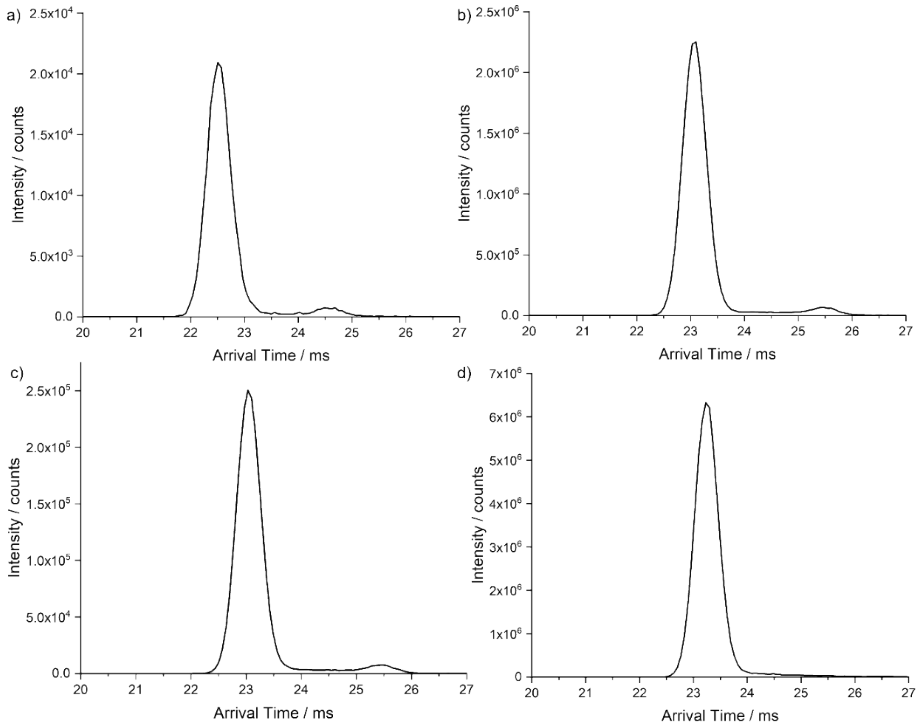

Figure S1. Arrival time distribution (ATD) of [M(AMPA)(AMPA-H)]$^+$ complexes with a) Mg^{2+}, b) Ca^{2+}, c) Sr^{2+}, and d) Ba^{2+} observed on the cIMS platform.

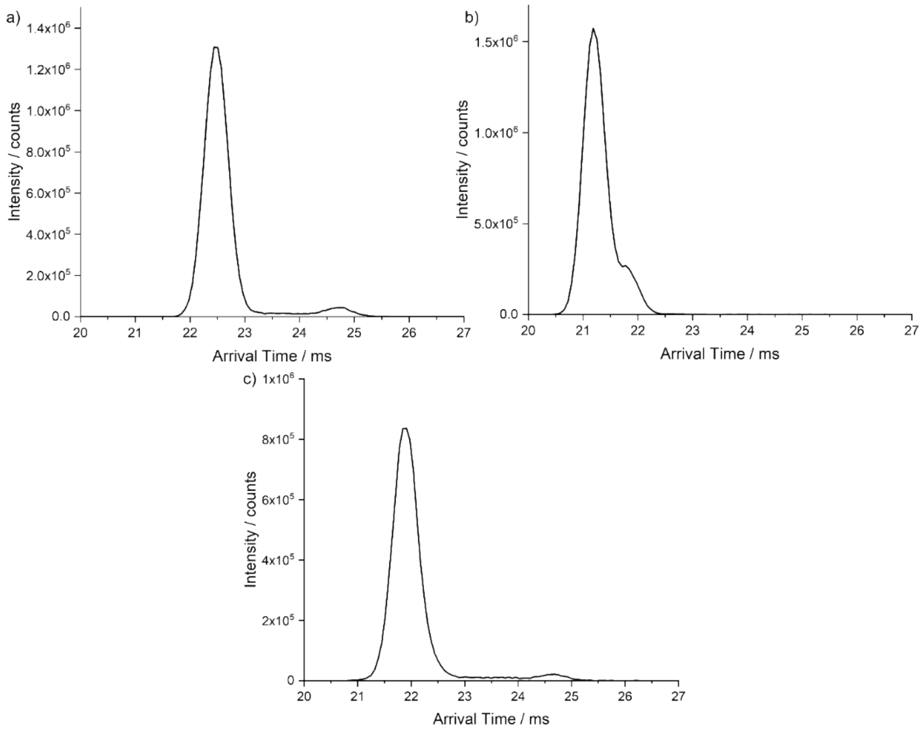

Figure S2. Arrival time distribution (ATD) of [M(AMPA)(AMPA-H)]$^+$ complexes with a) Mn^{2+}, b) Cu^{2+}, and c) Zn^{2+} observed on the cIMS platform.

Arrival Time Distribution of Mass-selected [M(AMPA)(AMPA-H)]$^+$ on TWIMS

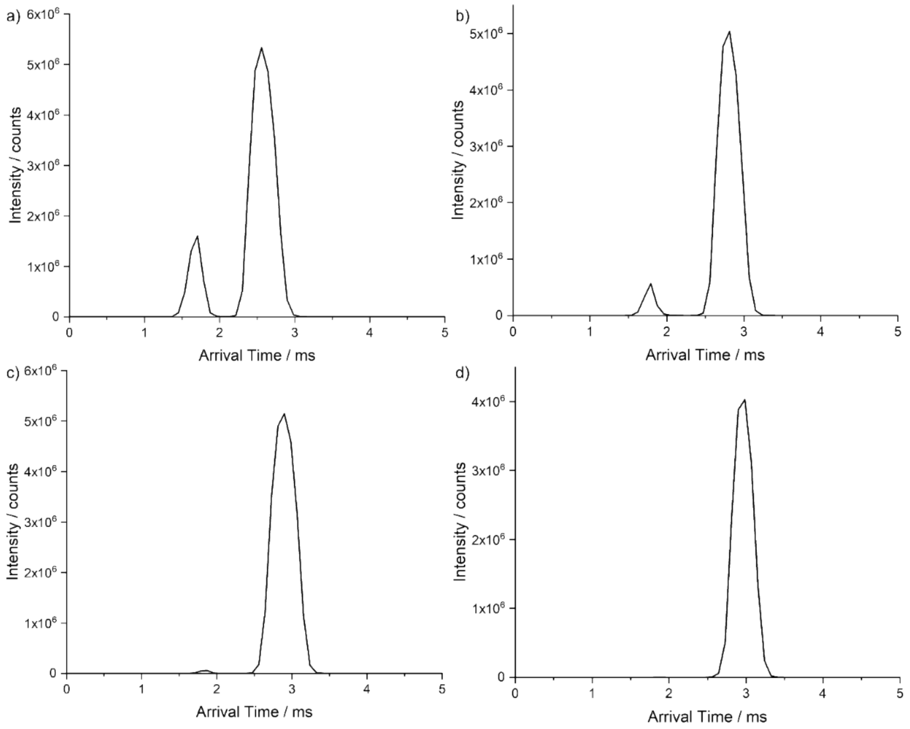

Figure S3. Arrival time distribution (ATD) of [M(AMPA)(AMPA-H)]$^+$ complexes with a) Mg^{2+}, b) Ca^{2+}, c) Sr^{2+}, and d) Ba^{2+} observed on the TWIMS(S) platform.

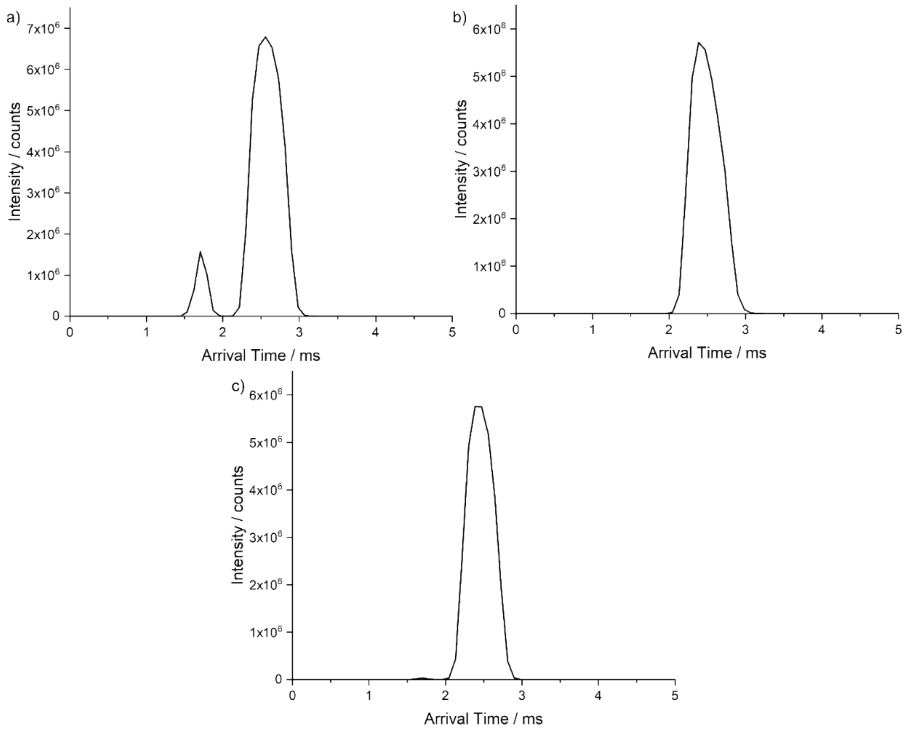

Figure S4. Arrival time distribution (ATD) of [M(AMPA)(AMPA-H)]$^+$ complexes with a) Mn^{2+}, b) Cu^{2+}, and c) Zn^{2+} observed on the TWIMS(S) platform.

Discussion: Extra Peaks from High Order (multiply charged) cluster fragmentation

Further investigation into the second peak observed on the mobilogram of $[Mg(AMPA)(AMPA\text{-}H)]^+$ and $[Ca(AMPA)(AMPA\text{-}H)]^+$ was conducted by extracting their mass spectra and comparing it to the extracted mass spectra of the most abundant peak (Figure A5). It was found that the target $[Mg(AMPA)(AMPA\text{-}H)]^+$ and $[Ca(AMPA)(AMPA\text{-}H)]^+$ complexes were present in the extracted mass spectra for the peaks that arrive earlier in the mobilogram, but with other unassigned peaks (Figure A5b and d). Unlike the mass spectra for the most abundant peaks (Figure A5a and c), there are other peaks that are more dominant than the mass-selected $[Mg(AMPA)(AMPA\text{-}H)]^+$ and $[Ca(AMPA)(AMPA\text{-}H)]^+$, even though these were mass-selected. These peaks are suspected to be fragments of multiply charged (higher order) assemblies (i.e. with the same m/z as the mass selected dimer) that have fragmented during the analysis. Since the $[Mg(AMPA)(AMPA\text{-}H)]^+$ and $[Ca(AMPA)(AMPA\text{-}H)]^+$ observed on these mass spectra are likely to be fragments from higher order assemblies, the mobility and CCS measured for those are not comparable with the singly charged, true dimer species. In the low-field equation, the charge of the ion is one of the variables that affects the mobility and CCS of an ion, thus accurate charge assignment is crucial in determining the CCS of an ion. Therefore, the available evidence suggests these additionally separated peaks of lower intensity are higher charge state isobars and are thus not investigated further here.

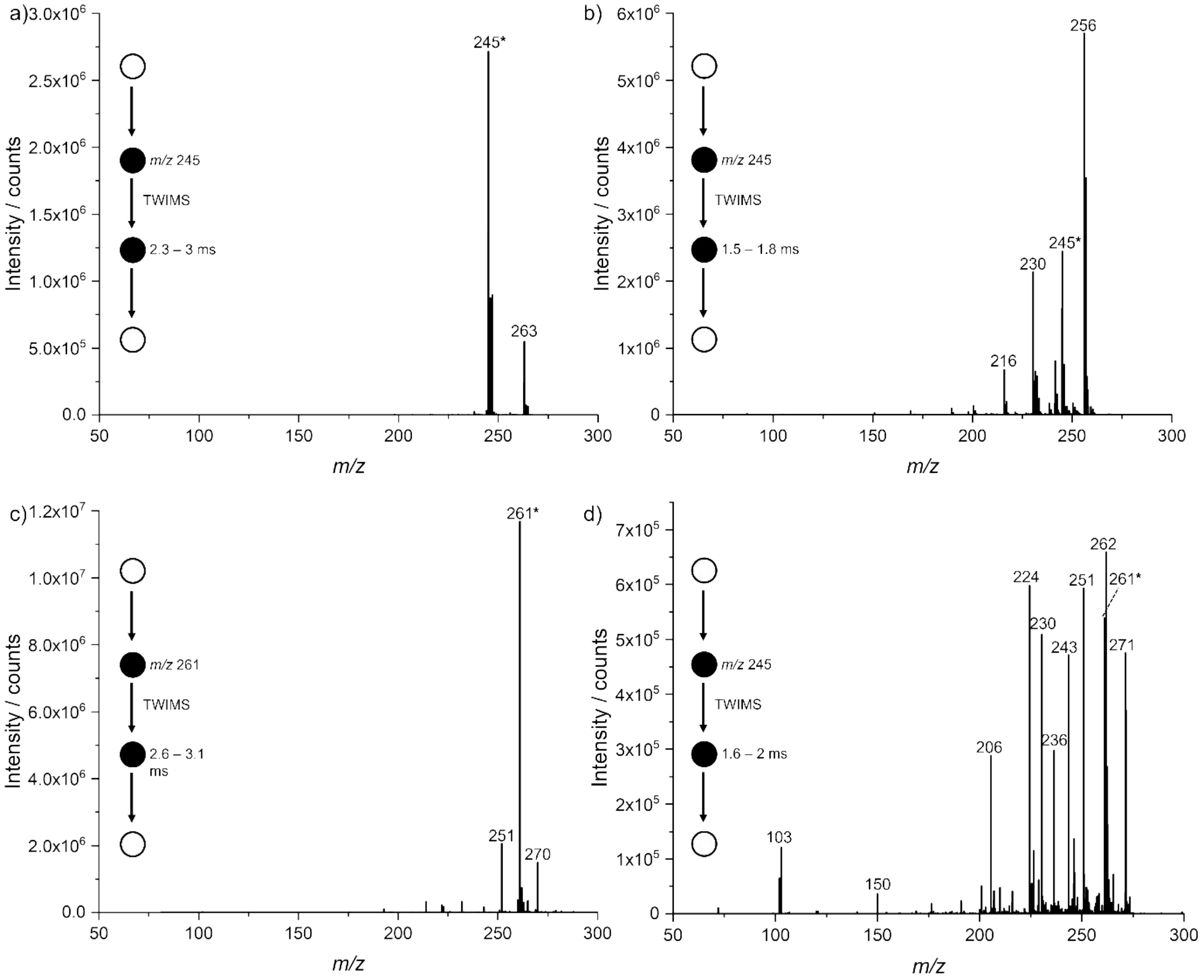

Figure S5. The mass-selected spectrum extracted from the mobilogram of [Mg(AMPA)(AMPA-H)]$^+$ (*m/z* 245) at a) 2.3-3 ms, b) 1.5-1.8 ms and [Ca(AMPA)(AMPA-H)]$^+$ (*m/z* 261) at c) 2.6-3.1 ms and d) 1.6-2 ms.

Discussion: Scaling Factors and the Comparison with Glyphosate Dimers

For all ${}^{Exp}CCS_{N2}$ values measured on both instruments, the difference between ${}^{Exp}CCS_{N2}$ and ${}^{Calc}CCS_{N2}$ (≤ 7%, Table S1) are smaller than that for $[M(glyphosate)(glyphosate-H)^{+}$ species (11-13%).[20] The ${}^{Calc}CCS_{N2}$ values still overestimate the ${}^{Exp}CCS_{N2}$ values, however to a smaller degree. For alkali earth metal complexes, the largest difference between ${}^{Exp}CCS_{N2}$ and ${}^{Calc}CCS_{N2}$ are for $[Sr(AMPA)(AMPA-H)]^{+}$ and $[Ba(AMPA)(AMPA-H)]^{+}$ at 5% and 7%, respectively which is larger than the difference for other $[M(AMPA)(AMPA-H)]^{+}$ complexes (Table S1). Even though for $[Cu(AMPA)(AMPA-H)]^{+}$ there was a shouldering peak observed on the cIMS platform, the difference between ${}^{Exp}CCS_{N2}$ and ${}^{Calc}CCS_{N2}$ for transition metal complexes is still less than 5% with the largest overestimation being ~5 Å^2 for the main peak of $[Cu(AMPA)(AMPA-H)]^{+}$.

Comparing with previous measured $[M(glyphosate)(glyphosate-H)]^{+}$ dimers,[20] the range in percentage difference between ${}^{Exp}CCS_{N2}$ and ${}^{Calc}CCS_{N2}$ for $[M(AMPA)(AMPA-H)]^{+}$ complexes is larger. This implies that the overestimation in ${}^{Calc}CCS_{N2}$ is less systematic and using a scaling factor more difficult. The current acceptable standard for structural assignment based on the comparison of ${}^{Exp}CCS_{N2}$ and ${}^{Calc}CCS_{N2}$ value is within ~2%. Thus, for $[M(AMPA)(AMPA-H)]^{+}$ complexes where the difference between the ${}^{Exp}CCS_{N2}$ and ${}^{Calc}CCS_{N2}$ values are within 2% (i.e. Mg^{2+}, Ca^{2+} and Mn^{2+}), the structures can be confidently assigned to the predicted global minima structures.

Focusing only on the ${}^{Calc}CCS_{N2}$ trend, when plotted against the metal cationic radius, the ${}^{Calc}CCS_{N2}$ values increase as the metal cationic radius increases, except for Cu^{2+} complex (Figure 1 a). This trend is similar to the one observed for $[M(glyphosate)(glyphosate-H)]^{+}$, [20] however, is not observed on the corresponding ${}^{Exp}CCS_{N2}$ values (Figure 1 a). This is again, evident from the seemingly small ${}^{Exp}CCS_{N2}$ values for $[Sr(AMPA)(AMPA-H)]^{+}$ and $[Ba(AMPA)(AMPA-H)]^{+}$. With regards to the application of an accurate scaling factor derived for $[M(glyphosate)(glyphosate-H)]^{+}$ complexes of 0.9 to the entirety of ${}^{Calc}CCS_{N2}$ for $[M(AMPA)(AMPA-H)]^{+}$, it can be seen that the scaled ${}^{Calc}CCS_{N2}$ values now underestimates the ${}^{Exp}CCS_{N2}$ by ~20 Å^2 and are a worse match to the measured ${}^{Exp}CCS_{N2}$ values (Table S1). This is crucial as a scaling factor should be applicable for a family of compound, however, in this case, the application of the same scaling factor resulted in a worse agreement between the ${}^{Exp}CCS_{N2}$ and ${}^{Calc}CCS_{N2}$. Therefore, for [M(AMPA)(AMPA-H)]+ complexes, the scaling factor is found to not be appropriate.

Table S2. a) $^{Exp}CCS_{N2}$ of $[M(AMPA)(AMPA\text{-}H)]^+$ measured on cIMS and TWIMS(S) platforms and b) the percentage difference to the corresponding $^{Calc}CCS_{N2}$ values calculated for the minimum structure. The standard deviation of the CCS measurement acquired in triplicate was ±0.1-0.3 Å^2 for both cIMS and TWIMS methods, corresponding to errors of ±0.1-0.2%).

	cIMS $^{Exp}CCS_{N2}$ / Å^2	Difference / %	TWIMS $^{Exp}CCS_{N2}$ / Å^2	Difference / %	Raw $^{Calc}CCS_{N2}$ / Å^2	Scaled $^{Calc}CCS_{N2}$ /Å^2
Mg^{2+}	151.8	2	152.7	1	154.5	139.1
Ca^{2+}	154.1	2	156.3	0	157.0	141.3
Sr^{2+}	152.9	5	156.6	3	160.8	144.8
Ba^{2+}	153.0	7	156.9	4	163.5	147.2
Mn^{2+}	150.7	2	151.8	1	153.1	137.8
Cu^{2+}	143.6	5	150.2	1	151.6	136.5
	146.9	3				
Zn^{2+}	147.4	4	149.8	3	154.1	138.7

Discussion: IRMPD Spectroscopy

To better understand the structure of $[Cu(AMPA)(AMPA-H)]^+$, the mass- and mobility-selected IR action spectrum was obtained and compared to the predicted IR spectrum for the global minimum structure. The experimental IR spectrum shows only one dominant peak at 3638 cm^{-1} , (Figure S6a), which can be assigned to the OH stretches on the phosphonate groups. The IR spectrum for the alternate structure (Figure S6c) has a strong NH absorption band ~ 3290 cm^{-1} that was not observed on the experimental spectrum. The structure of $[Cu(AMPA)(AMPA-H)]^+$ can be thus confidently assigned to the energetically preferred global minimum structure based on the spectral match and its CCS (See below for discussion on the strong absorption at ~2700 cm^{-1} in Figure S6b).

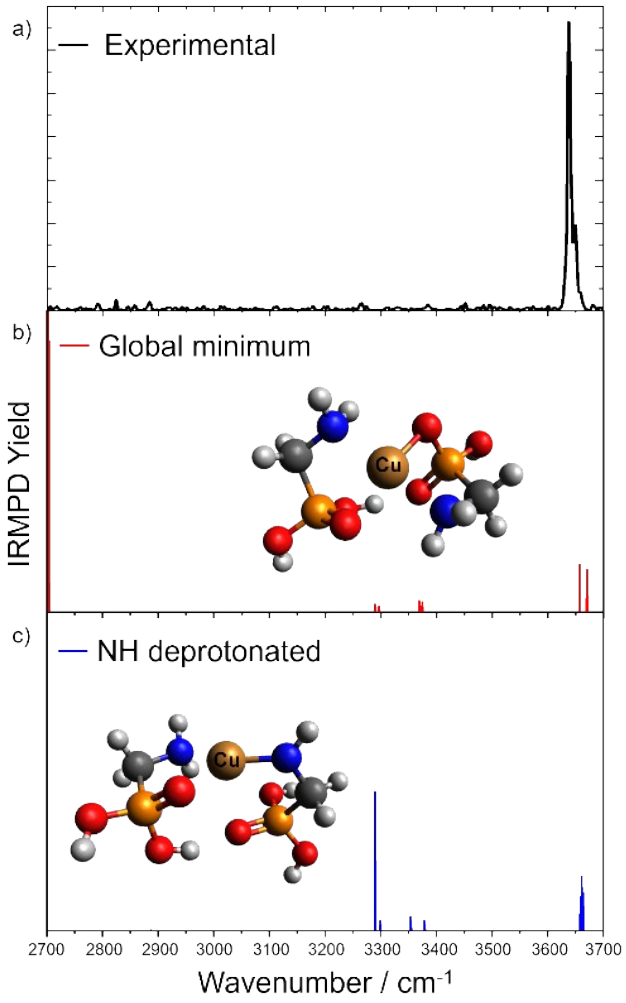

Figure S6. Comparison between a) experimental IR action spectrum and calculated IR spectra for b) the global minimum structure and c) an alternate structure for $[Cu(AMPA)(AMPA-H)]^+$ complex.

In the experimental spectrum, only 1 peak was observed at 3638 cm^{-1} (Figure S6). This peak has a matching predicted peak which can be assigned to the OH stretches on the phosphonate groups. This is interesting and consistent with the IR spectra observed for $[M(glyphosate)(glyphosate)]^+$ complexes

having two dominant peaks at 3550 and 3660 cm^{-1}, corresponding to the OH stretches on the phosphonate and carboxylate group.[20] AMPA does not have carboxylate groups, therefore only 1 peak is present in the IR spectrum (Figure S6 a).

In the predicted IR spectrum for the global minimum structure, there are peaks at 3660 cm^{-1} and an intense peak at ~2700 cm^{-1} that can be assigned to the OH-H bonding interaction (Figure S6 b), however, this peak wasn't observed experimentally. It is suspected that similar to [Cu(glyphosate)(glyphosate-H)]$^+$, the absence of the peak at 2700 cm^{-1} from the experimental spectrum is most likely caused by the strongly hydrogen bonded OH stretch resulting in a peak that is heavily broadened and diffuse. The strong hydrogen bonding interaction might have also resulted in shifting of the band outside of the IRMPD experiment.[21,22] The experimental IR spectrum was also compared to a predicted spectrum for an alternate [Cu(AMPA)(AMPA-H)]$^+$ structure, where the metal cation is bonded to the deprotonated NH group instead. This structure is ~40 kJ mol^{-1} higher in energy than the global minimum structure, and the predicted IR spectrum from this structure shows an intense peak at 3300 cm^{-1} that wasn't observed experimentally (Figure S6 c). Therefore, the structure of [Cu(AMPA)(AMPA-H)]$^+$ can be confidently assigned to the predicted global minimum structure due to the better agreement in experimental and predicted IR spectra and from energetical point of view.

Table S3. Theoretical IR frequency and their intensity for the global minimum structure and the alternate NH-deprotonated structure of [Cu(AMPA)(AMPA-H)]$^+$.

Global Minimum Structure			NH Deprotonated Structure		
**Wavenumber / cm^{-1}**	**Scaled Wavenumber / cm^{-1}**	**IR Intensity**	**Wavenumber / cm^{-1}**	**Scaled Wavenumber / cm^{-1}**	**IR Intensity**
2845.787	2703.497	1763.197	2976.780	2827.941	2.045
3070.881	2917.337	2.259	3039.001	2887.050	4.925
3076.737	2922.900	1.613	3064.261	2911.048	3.029
3134.506	2977.781	0.923	3126.958	2970.610	1.204
3144.803	2987.563	0.194	3463.396	3290.226	769.698
3462.959	3289.811	51.179	3473.409	3299.739	56.594
3470.265	3296.752	40.260	3530.655	3354.122	79.531
3546.046	3368.743	71.025	3556.549	3378.721	56.386
3552.072	3374.468	63.790	3851.514	3658.938	190.447
3849.034	3656.583	280.912	3854.066	3661.363	299.038
3863.334	3670.167	253.558	3856.594	3663.764	210.551

References

1 O. H. Lloyd Williams and N. J. Rijs, *Front. Chem.*, DOI:10.3389/fchem.2021.682743.
2 O. Rusli, O. H. L. Williams, P. Chakraborty, M. Neumaier, F. Hennrich, S. Bakels, K. Hes, A. M. Rijs, B. Ucur, S. R. Ellis, R. J. Pachulicz, T. L. Pukala and N. J. Rijs, *Phys. Chem. Chem. Phys.*, 2025, **27**, 7519–7531.
3 M. F. Bush, I. D. G. Campuzano and C. V. Robinson, *Anal. Chem.*, 2012, **84**, 7124–7130.
4 F. Hennrich, S. Ito, P. Weis, M. Neumaier, S. Takano, T. Tsukuda and M. M. Kappes, *Phys. Chem. Chem. Phys.*, 2024, **26**, 8408–8418.
5 S. Bakels, S. Daly, B. Doğan, M. Baerenfaenger, J. Commandeur and A. M. Rijs, *Anal. Chem.*, DOI:10.1021/acs.analchem.4c02749.
6 F. Neese, *WIREs Computational Molecular Science*, 2012, **2**, 73–78.
7 F. Neese, F. Wennmohs, U. Becker and C. Riplinger, The ORCA quantum chemistry program package | The Journal of Chemical Physics | AIP Publishing, https://pubs.aip.org/aip/jcp/article/152/22/224108/1061982/The-ORCA-quantum-chemistry-program-package, (accessed February 11, 2025).
8 M. J. Frisch, G. W. Trucks, H. B. Schlegel, G. E. Scuseria, M. A. Robb, J. R. Cheeseman, G. Scalmani, V. Barone, G. A. Petersson, H. Nakatsuji, X. Li, M. Caricato, A. V. Marenich, J. Bloino, B. G. Janesko, R. Gomperts, B. Mennucci, H. P. Hratchian, J. V. Ortiz, A. F. Izmaylov, J. L. Sonnenberg, Williams, F. Ding, F. Lipparini, F. Egidi, J. Goings, B. Peng, A. Petrone, T. Henderson, D. Ranasinghe, V. G. Zakrzewski, J. Gao, N. Rega, G. Zheng, W. Liang, M. Hada, M. Ehara, K. Toyota, R. Fukuda, J. Hasegawa, M. Ishida, T. Nakajima, Y. Honda, O. Kitao, H. Nakai, T. Vreven, K. Throssell, J. A. Montgomery Jr., J. E. Peralta, F. Ogliaro, M. J. Bearpark, J. J. Heyd, E. N. Brothers, K. N. Kudin, V. N. Staroverov, T. A. Keith, R. Kobayashi, J. Normand, K. Raghavachari, A. P. Rendell, J. C. Burant, S. S. Iyengar, J. Tomasi, M. Cossi, J. M. Millam, M. Klene, C. Adamo, R. Cammi, J. W. Ochterski, R. L. Martin, K. Morokuma, O. Farkas, J. B. Foresman and D. J. Fox, Gaussian 16 2016.
9 P. Pracht, F. Bohle and S. Grimme, *Phys. Chem. Chem. Phys.*, 2020, **22**, 7169–7192.
10 S. Grimme, F. Bohle, A. Hansen, P. Pracht, S. Spicher and M. Stahn, *J. Phys. Chem. A*, 2021, **125**, 4039–4054.
11 P. Pracht, S. Grimme, C. Bannwarth, F. Bohle, S. Ehlert, G. Feldmann, J. Gorges, M. Müller, T. Neudecker, C. Plett, S. Spicher, P. Steinbach, P. A. Wesołowski and F. Zeller, *J Chem Phys*, 2024, **160**, 114110.
12 C. Bannwarth, S. Ehlert and S. Grimme, GFN2-xTB—An Accurate and Broadly Parametrized Self-Consistent Tight-Binding Quantum Chemical Method with Multipole Electrostatics and Density-Dependent Dispersion Contributions | Journal of Chemical Theory and Computation, https://pubs.acs.org/doi/10.1021/acs.jctc.8b01176, (accessed February 12, 2025).
13 H. Mun, W. Lorpaiboon and J. Ho, *J. Phys. Chem. A*, 2024, **128**, 4391–4400.
14 O. Rusli, O. H. Lloyd Williams, P. Chakraborty, M. Neumaier, F. Hennrich, S. Bakels, K. Hes, A. M. Rijs, B. Ucur, S. R. Ellis, R. J. Pachulicz, T. L. Pukala and N. J. Rijs, *Phys. Chem. Chem. Phys.*, DOI:10.1039/D4CP04019H.
15 I. M. Alecu, J. Zheng, Y. Zhao and D. G. Truhlar, *J. Chem. Theory Comput.*, 2010, **6**, 2872–2887.
16 F. Weigend and R. Ahlrichs, *Phys. Chem. Chem. Phys.*, 2005, **7**, 3297–3305.
17 L. G. Migas, C. J. Gray, S. L. Flitsch and P. E. Barran, *A Careful Consideration of the Influence of Structure, Partial charges and Basis Sets on Collision Cross Sections of Monosaccharides when Comparing Values from DFT Calculated Conformers to those Obtained Experimentally*, 2017.
18 C. Larriba-Andaluz and C. J. Hogan Jr., *The Journal of Chemical Physics*, 2014, **141**, 194107.
19 M. F. Mesleh, J. M. Hunter, A. A. Shvartsburg, G. C. Schatz and M. F. Jarrold, *J. Phys. Chem.*, 1996, **100**, 16082–16086.

20 O. Rusli, S. Bakels, K. Hes, H. Mun, O. H. Lloyd Williams, R. J. Pachulicz, T. L. Pukala, M. Neumaier, F. Hennrich, J. Ho, A. M. Rijs and N. J. Rijs, *J. Am. Soc. Mass Spectrom.*, 2025, **36**, 1296–1307.
21 J. Spanget-Larsen, B. K. V. Hansen and P. E. Hansen, *Chemical Physics*, 2011, **389**, 107–115.
22 P. E. Hansen and J. Spanget-Larsen, *Journal of Molecular Structure*, 2012, **1018**, 8–13.
